# Immunogenetic Background of Chronic Lymphoproliferative Disorders in Romanian Patients—Case Control Study

**DOI:** 10.3390/medsci12010014

**Published:** 2024-02-23

**Authors:** Maria Tizu, Bogdan Calenic, Ion Maruntelu, Andreea Mirela Caragea, Adriana Talangescu, Larisa Ursu, Corina Rotarescu, Mariana Surugiu, Alexandra Elena Constantinescu, Ileana Constantinescu

**Affiliations:** 1 Immunology and Transplant Immunology, Carol Davila University of Medicine and Pharmacy, 258 Fundeni Avenue, 022328 Bucharest, Romania; bcalenic@yahoo.co.uk (B.C.); corina.rotarescu@drd.umfcd.ro (C.R.);; 2 Centre of Immunogenetics and Virology, Fundeni Clinical Institute, 258 Fundeni Avenue, 022328 Bucharest, Romaniaadriana.oprea@drd.umfcd.ro (A.T.); 3 Academy of Romanian Scientists (AOSR), 3 Ilfov Street, Sector 5, 022328 Bucharest, Romania

**Keywords:** chronic lymphoproliferative disorders, human leukocyte antigens (HLAs), peripheral T-cell lymphoma not otherwise specified, Burkitt lymphoma, diffuse large B-cell lymphoma

## Abstract

Background and Objectives: The implications of the genetic component in the initiation and development of chronic lymphoproliferative disorders have been the subject of intense research efforts. Some of the most important genes involved in the occurrence and evolution of these pathologies are the HLA genes. The aim of this study is to analyze, for the first time, possible associations between chronic lymphoproliferative diseases and certain HLA alleles in the Romanian population. Materials and Methods: This study included 38 patients with chronic lymphoproliferative disorders, diagnosed between 2021 and 2022 at Fundeni Clinical Institute, Bucharest, Romania, and 50 healthy controls. HLA class I and class II genes (HLA-A/B/C, HLA-DQB1/DPB1/DRB1) were investigated by doing high resolution genotyping using sequence specific primers (SSP). Results: Several HLA alleles were strongly associated with chronic lymphoproliferative disorders. The most important finding was that the HLA-C*02:02 (*p* = 0.002, OR = 1.101), and HLA-C*12:02 (*p* = 0.002, OR = 1.101) have a predisposing role in the development of chronic lymphoproliferative disorders. Moreover, we identified that HLA-A*11:01 (*p* = 0.01, OR = 0.16), HLA-B*35:02 (*p* = 0.037, OR = 0.94), HLA-B*81:01 (*p* = 0.037, OR = 0.94), HLA-C*07:02 (*p* = 0.036, OR = 0.34), HLA-DRB1*11:01 (*p* = 0.021, OR = 0.19), and HLA-DRB1*13:02 (*p* = 0.037, OR = 0.94), alleles have protective roles. Conclusions: Our study indicates that HLA-C*02:02 and HLA-C*12:02 are positively associated with chronic lymphoproliferative disorders for our Romanian patients while HLA-DRB1*11:01, HLA-DRB1*13:02, and HLA-B*35:02 alleles have a protective role against these diseases.

## 1. Introduction

Lymphoproliferative disorders (LPDs) are a group of diseases with diverse manifestations but that are characterized mainly by uncontrolled production of monoclonal lymphoid cells [1,2]. Traditionally clinicians divided lymphoproliferative diseases into three categories: leukemias or white blood cell cancers, lymphomas or lymphocytic malignancies that produce solid tumors, and monoclonal gammopathies, which were characterized as malignant proliferations of B lymphocytes and plasma cells [3].

According to the fifth edition of the WHO Classification, hematopoietic tumors and lymphoid tumors are divided in three distinct categories, B-cell lymphoid proliferations and lymphomas, T-cell and NK-cell lymphoid proliferations, and lymphomas and stroma-derived neoplasms of lymphoid tissues [4].

Chronic lymphoproliferative diseases preserve the heterogeneous character of the main group of lymphoproliferative diseases. They are defined by an increased proliferation of lymphocytes [1] and also by the absence of terminal deoxynucleotidyl transferase [5], an important marker in differentiating between acute and mature cell malignancies [6]. In 2016, the WHO classified mature lymphoid, histiocytic, and dendritic neoplasms as mature B-cell neoplasms, mature T and NK neoplasms, Hodgkin lymphoma, Posttransplant lymphoproliferative disorders (PTLD), and histiocytic and dendritic cell neoplasms [7]. While it is widely recognized that certain LPDs exhibit a significant autoimmune component, the primary focus of the current study was on chronically malignant LPDs rather than those associated with autoimmunity [1].

The latest WHO classification of hematolymphoid disorder underlines the increased importance of the genetic factor in the evaluation of lymphoid neoplasms. The human leukocyte antigen complex is the homologous of the major histocompatibility complex [MHC] in humans [8]. Because of their crucial involvement in the immune system and their polymorphic features [8], these genes have been the focus of intense research. Their distinctive ability to discern between ‘self’ and ‘non-self’ antigens ensures the continuous protection of the body against pathogens [9,10,11]. The HLA genes manage to do this by encoding MHC class I and II molecules [11,12].

It is well known that MHC class I molecules are present in the majority of nucleated cells, while MHC class II molecules are specifically found in certain cells, particularly antigen-presenting cells [9,10,12]. Together, MHC class I and II molecules intervene in the presentation of antigens to T lymphocytes, this being an essential step in the activation of these cells and in the initiation of an immune response [11,13,14].

In order to differentiate between various pathogens, MHC molecules need to exhibit significant diversity [15]. This diversity is facilitated by the extensive polymorphism of the HLA genes, encompassing hundreds of alleles that encode a wide array of MHC molecules. These diverse MHC molecules can effectively withstand the diversity of microorganisms encountered throughout our lives [15,16].

Another distinctive feature of HLA genes is their remarkable variability across populations [17]. This trait has been extensively studied, leading to the creation of databases that document alleles found in specific regions of the world, providing insights into the most prevalent allelic variants among diverse populations [17].

The unique roles and characteristics of these genes have prompted researchers to look for associations with a wide range of pathologies, like different types of cancers, gastro-intestinal diseases, autoimmune diseases, or viral infections [18,19]. The genetic component of chronic lymphoproliferative diseases was also underlined in several studies such as Wang et al., Takeuchi et al., or Luo et al. [20,21,22]. Thus, some of the genes most commonly associated with the occurrence and prognosis of these diseases are HLA genes. Previous studies mention the predisposing role of HLA-B*08:01, HLA-DRB1*03:01, and HLA-DRB1*09 alleles in the case of diffuse large B-cell lymphoma [23,24,25]. Other research papers debate the importance and the involvement of HLA genes in the occurrence of several pathologies encountered less frequently; HLA-C*07 was identified as a predisposing factor for adult T-cell lymphoma [26], while HLA DR5 was positively associated with Mycosis fungoides [27,28], and HLA DRB1*11:04 increases the risk of developing Sézary syndrome [28,29]. While these findings are promising, the challenge of establishing associations between genes and diseases universally valid for all populations persists, primarily due to the considerable variability of HLA genes.

The variables involved in defining the protective or the predisposing roles of HLA genes associated with chronic lymphoproliferative disorders include patient demographic parameters, severity of the disease, or geographical region where the study took place. To date, there are no studies focusing on the connection between HLA and LPD in Romanian patients. In this context, the specific aim of the present article is to explore for the first-time potential associations of HLA and chronic lymphoproliferative disorders in Romanian patients.

## 2. Materials and Methods

### 2.1. Patients and Controls

For this study, we selected 38 patients with a variety of with lymphoproliferative diseases with mature cell, that were diagnosed in the Department of Hematology at Fundeni Clinical Institute. We took into account the pathologies classified by the World Health Organization (WHO) as mature lymphoid neoplasms [7]. Patients with peripheral T-cell lymphoma not otherwise specified (PTCL-NOS, 16 cases), Burkitt lymphoma (5 cases), diffuse large B-cell lymphoma (DLBCL, 6 cases), adult T-cell lymphoma (ATLL, 4 cases), primary cutaneous γδ T-cell lymphoma (2 cases), mantle cell lymphoma (3 cases), mycosis fungoides (1 case), and Sézary syndrome (1 case) were included in the study. To date, epidemiological data on LPDs in the Romanian population is virtually non-existent [30].

Several key diagnostic parameters were considered, including blood cell counts, peripheral blood smear with evidence of cell morphology, and immunophenotypic analysis. Using ESMO Clinical Practice Guidelines, the patients were diagnosed between 2021 and 2022 [31]. From an initial total of 64 patients, only 38 meat the eligibility criteria and were admitted in the study. These criteria included being able to provide direct informed consent or through a legal guardian, free from pregnancy, active infections, central nervous system disorders, cardiovascular diseases, severe lung diseases, severe kidney diseases, severe allergies, severe autoimmune diseases, and the absence of other associated cancers or diseases. The exclusion criteria also encompassed mental disorders that could interfere with study participation, positive serology for B and C Hepatitis, and additionally, for each blood donor volunteer participating in the study, their medical history was examined from the medical personal file, and biochemical parameters and viral status were assessed in compliance with national requirements during the blood donation procedure.

Following diagnosis, all patients received appropriate chemotherapy with variations tailored to the specific pathology and individual progression of each patient. So, the 6 patients diagnosed with DLBCL underwent R-CHOP treatment, which includes rituximab, cyclophosphamide, adriamycin, vincristine, and prednisone. Conversely, for individuals with Burkitt’s lymphoma, the preferred regimen was R-hyper-CVAD (rituximab, cyclophosphamide, vincristine, adriamycin, and dexamethasone). Patients with T-cell LPD were administered the classic CHOP treatment. Notably, two patients with PTCL-NOS, in addition to cyclophosphamide, vincristine, and prednisone, also received epirubicin (CEOP).

The patients group consisted of subjects between the ages 16 and 65 years with a median age of 45.47. Out of 38 patients, 13 were female (34.2%) and 25 patients were male (65.8%). We calculated the median age through descriptive statistics using SPSS software version 28.0. Our study cohort exhibited a gender distribution consistent with the numbers encountered in the existing literature, demonstrating a double occurrence rate among men (male:female ratio approximately 2:1) [1,32]. However, in contrast to the literature, the median age in our study group was lower than the conventional median age of 65 years typically associated with the onset of these pathologies [32]. The lowest median age encountered was, as expected, in patients with Burkitt lymphoma, while patients with PTCL and DLBCL were older, with a median age of 50 and 44, respectively.

The control group comprised 50 bone marrow donors included in the National Bone Marrow Donor Registry, 22 female and 28 male (see Table 1). To avoid bias, the controls included in the study were not related to the patients in the study group.

HLA typing was performed for all patients within the initial year following diagnosis Among our cohort of 16 PTCL-NOS patients, 4 underwent autologous hematopoietic cell transplantation (auto-HCT), and of these, 2 successfully survived. From the group of 6 patients diagnosed with DLBCL, 2 underwent autologous hematopoietic cell transplantation (auto-HCT) and achieved sustained survival. Within the subset of 5 patients with Burkitt lymphoma, 2 underwent autologous hematopoietic cell transplantation (auto-HCT); however, only one exhibited a survival duration surpassing one year and continues to live at present.

This study was approved by The Ethical Committee of Fundeni Clinical Institute no. 46893. Written consent was collected from both patient and control groups in accordance with the Declaration of Helsinki.

### 2.2. Sample Collection and DNA Extraction

For DNA extraction, 5 mL of whole blood was collected from each patient and each control on EDTA or citrate tubes. The extraction kit used was a QIAmp DNA Blood Mini^®^ kit (QIAGEN, Hilden, Germany). DNA was extracted from 200 µL of whole blood using a silica membrane. After purifying the DNA, it was separated from the silica membrane using an elution buffer and collected in separate tubes. The DNA was stored at −18 °C until use. We determined the DNA purity and concentration using an IMPLEN nanophotometer (Westlake Village, CA, USA), considering acceptable all samples with a DNA concentration > 20 ng/µL and a purity between 1.7 and 1.9.

### 2.3. HLA Analysis

To investigate HLA gene polymorphisms, we performed HLA high resolution genotyping using sequence-specific primers (SSP). With AllSet+™ Gold SSP (Invitrogen, Carlsbad, CA, USA) kits, we have typed class I (HLA-A/B/C) and class II (HLA-DQB1/DPB1/DRB1) genes. This HLA typing method utilizes multiple pairs of cis-located allele-specific primers to identify the alleles within a given DNA sample. Sequence-specific primers (SSPs) are assays designed to bind to and amplify polymorphic regions.

Each locus is associated with a dedicated amplification plate featuring a varying number of strips/wells. Specifically, plates with 12 strips (96 wells) are employed for HLA class I genes and HLA-DRB1, 6 strips for HLA-DPB1, and 4 strips for HLA-DQB1. These plates incorporate amplification primers, with each plate having a well designated with a black band corresponding to the negative control, positioned on strip 1 in the H well.

An amplification mix was added in each of these plates as follows. A mix containing PCR Buffer, TAq polymerase, and water WAs prepared for the well corresponding to the negative control. For the remaining wells, the mix additionally included DNA. As a general guideline, 66 µL of PCR Buffer, 1 µL of Taq, 87 µL of water, and 18 µL of DNA were added for each strip, and this composition was multiplied by the number of strips associated with each locus.

After pipetting 10 µL of the mixture into each well, the plates went into the PCR for amplification. Post-PCR processing requires agarose gel electrophoresis to highlight, the obtained amplification products. The analyzed gel image revealed a control band in every well, and additionally, certain wells exhibited a specific allele band referred to as an amplicon.

The analysis of reaction patterns was performed automatically with the help of the UniMatch^®^ version 6.0 software which compared the results with the IMGT/HLA international database updated to the current date for the most precise interpretation.

### 2.4. Statistical Analysis

We conducted a case–control study in which we used IBM^®^ SPSS^®^ Statistics software (Chicago, IL, USA) version 28, 2022, to identify associations between various HLA alleles and chronic lymphoproliferative disorders. To determine the median age, we used descriptive statistics. The Chi-square test or Fisher’s exact test was employed to determine HLA–disease association. Odds ratios (ORs) with 95% confidence intervals (CIs) were computed to assess the strength of associations, with statistical significance indicated by *p* < 0.05.

## 3. Results

We analyzed HLA genes in patients with lymphoproliferative disorders and compared them with the control group. We examined both haplotypes for patients and controls, which resulted in 76 alleles being analyzed in the patient’s group and 100 alleles in the control group.

Using SSP genotyping, we evaluated HLA class I (HLA-A/B/C) and class II (HLA-DPB1/DQB1/DRB1) genes with the purpose of finding possible statistical connections between these genes and lymphoproliferative disorders. We collected detailed results for both groups. We identified 26 HLA-A alleles, 38 HLA-B alleles, 25 HLA-C alleles, 16 HLA-DPB1 alleles, 16 HLA-DQB1 alleles, and 28 HLA-DRB1 alleles.

We wanted to identify possible associations between HLA genes and chronic lymphoproliferations as a whole. So, we assessed allele frequencies at the four-digit level and identified six protective and two predisposing alleles for lymphoproliferative disorders. We identified HLA-A*11:01 (*p* = 0.010, OR = 0.169), HLA-B*35:02 (*p* = 0.037, OR = 0.940), and HLA-B*81:01 (*p* = 0.037, OR = 0.940) as having a strong protective role (see Table 2).

A key finding of our study was the connection we established between HLA-C genes and lymphoproliferative disorders. In these groups of genes, we identified two strong associations between HLA-C*02:02 (*p* = 0.002, OR = 1.101) and HLA-C*12:02 (*p* = 0.002, OR = 1.101) and lymphoproliferative disorders. Also, we established a protective role of HLA-C*07:02 (*p* = 0.036, OR = 0.345) (see Table 2).

Although HLA class II genes are represented less, we identified two alleles with a strong protective role, both from HLA-DRB1 genes. HLA-DRB1*11:01 (*p* = 0.021, OR = 0.190) and HLA-DRB1*13:02 (*p* = 0.037, OR = 0.940) were well expressed in controls, as shown in Supplementary Table S1.

We also evaluated the frequency of HLA alleles with a statistically proven impact on the most common pathologies: PTLC-NOS, DLBCL, Burkitt lymphoma, and ATLL. Of the 38 patients with chronic lymphoproliferative disorders, 16 patients were diagnosed with peripheral T-cell, 5 patients with Burkitt lymphoma, 6 patients with diffuse large B-cell lymphoma, and 4 patients with adult T-cell lymphoma. For comparison, we compared each group of patients with the same 50 controls formerly described.

For the PTLC-NOS population we identified one allele associated with the disease HLA-C*12:02 (*p* = 0.0001, OR = 1.231) and one allele with a protective role: HLA-A* 11:01 (*p* = 0.009, OR = 0.128, Supplementary Table S2). In the case of the DLBCL group, we encountered one protective allele: HLA-B*39:01 (*p* = 0.003, OR = 0.06, Supplementary Table S3). For patients with Burkitt lymphoma, we discovered HLA-C*06:02 (*p* = 0.047, OR = 0.233) for its protective role, as can be seen in Table 3. For the ATLL group of patients, no statistically significant results were established.

## 4. Discussion

Certain HLA alleles are already recognized as disease markers for specific pathologies [33,34,35,36]. The most relevant example is the association between HLA-B*27 and ankylosing spondylitis [33]. The presence of this allele not only assists clinicians in tailoring treatment but also signals a susceptibility within the patient’s family, warranting rigorous monitoring [33,34]. Similarly, the presence of HLA-DQ2 and HLA-DQ8 elevates the risk of celiac disease in carrier populations [35].

Numerous associations between HLA genes and different chronic lymphoproliferative disorders have been confirmed over the years [37,38,39,40,41]. Nevertheless, to the best of our knowledge, there have not been published any reports on the Romanian population on this matter. Motivated by these established connections, we explored potential links between chronic lymphoproliferations and HLA alleles. Our aim was to analyze the broader association between these genes and chronic lymphoproliferations as a whole. At the same time, we assessed specific associations of these genes with PTCL-NOS, Burkitt lymphoma, DLBCL, ATLL, primary cutaneous γδ T-cell lymphoma, mantle cell lymphoma, mycosis fungoides, and Sézary syndrome, recognizing the distinct significance of each within our research.

The classification of lymphoid neoplasms by the World Health Organization (WHO) has been recently updated to include considerations of both tumor morphology and genetic factors [4]. Essential attributes of several diseases, some included in the present study, are briefly discussed below. T-Cell lymphoma (PTCL) is a rare, aggressive type of non-Hodgkin’s lymphoma (NHL) originating from mature lymphocytes, making up 10–20% of all NHL cases [42,43]. The World Health Organization’s 2016 classification lists over 20 PTCL subtypes, with PTCL not otherwise specified (PTCL-NOS), angioimmunoblastic T-cell lymphoma, and anaplastic large cell lymphoma being the most common [42]. Burkitt lymphoma (BL) is a fast-growing type of B-cell non-Hodgkin lymphoma that originates from germinal center B-cells [44]. The World Health Organization (WHO) grouped BL into three categories: endemic, sporadic, and immunodeficiency-related [45]. Recent updates have removed certain categories, including the previously unclassifiable “B-cell lymphoma” that was identified by “high-grade B-cell lymphoma, with MYC and BCL-2 and/or BCL-6 rearrangements” [46]. Moreover, classifications that used to be under “Burkitt-like” lymphoma are now categorized as either high-grade B-cell lymphoma with MYC and BCL-2 and/or BCL-6 rearrangement, Burkitt-like lymphoma with 11q aberration, or simply high-grade B-cell lymphoma [46]. Diffuse large B-cell lymphoma (DLBCL) represents the most prevalent form of non-Hodgkin lymphomas (NHL), making up 30–40% of all B-cell NHL cases [47]. In the fifth edition of the WHO classification of lymphomas, over 80% of DLBCL cases fall under the category “not otherwise specified” (NOS), indicating they are a diverse collection of lymphomas without the specific diagnostic features of particular large B-cell lymphoma types [47,48]. The WHO-HAEM5 and the International Consensus Classification (ICC) both characterize adult T-cell leukemia/lymphoma (ATLL) as a type of peripheral T-cell cancer originating from CD4-positive T-cells infected with human T-cell leukemia virus (HTLV) type 1 [49,50]. Typical symptoms of ATLL include immunosuppression, lesions on the skin and bones, enlarged liver (hepatomegaly), swollen lymph nodes (lymphadenopathy), enlarged spleen (splenomegaly), and elevated calcium levels in the blood (hypercalcemia) [49]. Also, according to WHO-HAEM5, several entities are included in the primary cutaneous γδ T-cell lymphoma classification such as mycosis fungoides, the most common cutaneous T-cell lymphoma; primary cutaneous Acral CD8+ T-cell lymphoproliferative disease; and primary cutaneous peripheral T-cell lymphoma not otherwise specified (NOS) [49].

Our group was the first one that has analyzed the HLA allele distribution and frequency in the Romanian population, underlying its clinical significance and medical utility [51,52]. In the last decade, several other Romanian researchers have analyzed different associations of HLA genes with several pathologies. One of the first studies was carried out by Guja et al. [53] who highlighted strong predisposing and protective associations of HLA-DQB1 in type 1 diabetes mellitus. Recently, our research group has identified HLA class I and II alleles that predispose patients to chronic renal failure [54] or increase the risk of developing hepatitis B [55]. In another work by Maruntelu et al. [56], they also showed that the occurrence of celiac disease is closely associated to the HLA-DQA1*05:01, HLA-DQB1*02:01, and HLA-DQB1*02:02 allele expression in Romanian patients. These results motivated us to study new associations of HLA genes with other pathologies in the Romanian population.

Among the most important findings of our study were two HLA class I alleles, HLA-C*02:02 and HLA-C*12:02, which were positively associated with our target pathologies. Particularly, HLA-C*06:02 was identified as having a protective role against Burkitt lymphoma, while HLA-C*12:02 was positively associated with PTLC-NOS. A study conducted by Zong et al. [37] discovered an allele from the HLA-C*12 group, namely HLA-C*12:03 that is part of a haplotype HLA-A*2601~C*1203~B*3801~DRB1*0402~DQB1*0302 positively associated with DLBCL in Caucasians. HLA-C*12:02 has also been documented for its association with psoriasis [57,58].

An important discovery is represented by the determination of the protective role of two HLA-B alleles, HLA-B*35:02 and HLA-B*81:01, for all patients with lymphoproliferative disorders, while we discovered that HLA-B*39:01 has a protective effect only against DLBCL. Analyzing the literature, we found multiple studies that, like us, record the protective role of HLA-B*35. In particular. Wang et al. [20] also discussed the lower risk of developing NHL in patients with HLA-B*35:03. Basaran et al. [59] highlight the protective role of HLA-B35 against neoplastic transformation in patients with mycosis fungoides and Sézary syndrome. On the other hand, Hojjat-Farsangi et al. [60] report a higher incidence of HLA-B*35:01 in patients with chronic lymphocytic leukemia. Also, Brazzelli et al. [39] noticed an association between HLA-B*35 and mycosis fungoides. Similar results were obtained by, Benencio et al. [26], who reported an increased susceptibility to develop myelopathy/tropical spastic paraparesis for patients infected with human T-cell lymphotropic virus type 1 (HTLV-1), which also have HLA-B*35 present. Another finding of the same report showed an increased susceptibility of HLA-C*07 patients to developed ATLL [26], data which contradict our findings that talk about the protective role of HLA-C*07:02. The conflicting outcomes could be due to the phylogenetic distance between our Romanian population and the Argentine population. Additionally, this association should be validated on larger groups of patients. Jeffery et al. [38] emphasize the protective role of HLA-Cw*08 in patients with HTLV-I-associated myelopathy. Wang et al. [61] mentioned the importance of HLA-C*07:02 in the evolution of multiple myeloma.

Another finding was the protective role of HLA-A*11:01, and especially against PTLC-NOS which was significantly expressed in controls. The same decreased risk of developing the disease was reported for Hodgkin lymphoma patients with HLA-A11 [28,62]. HLA-A*11 was also found by Gavioli et al. [63] to have an important down-regulating role in the case of Burkitt lymphoma. A study among Koreans noted the protective role of HLA-A*33 against DLBCL [24].

Our findings indicate that HLA class II genes were represented the best by HLA-DRB1 genes. We have identified two HLA-DRB1 alleles, HLA-DRB1*11:01 (*p* = 0.021, OR = 0.190) and HLA-DRB1*13:02 (*p* = 0.037, OR = 0.940) which were expressed in the control group but not in patients indicating a protective effect. Our results are also supported by Wang et al. [20] who identified HLA-DRB1*13 as having a protective role against follicular lymphoma. In the case of mycosis fungoides and Sezary syndrome, this protective association was mentioned by Jackow et al. [27], who identified HLA-DR6 (*1301, *1302, and *1402) but unfortunately, the association did not remain valid after the correction of *p*. Another study conducted by Galleze et al. [64] mentioned HLA-DRB1*13 and HLA-DQB1*03 for their protective effect against Hodgkin lymphoma and non-Hodgkin lymphoma.

The same role of this group of alleles was determined also for Finns with multiple sclerosis [65], multiple populations with rheumatoid arthritis [66,67], and for Iranians with hepatitis B viral infection [68]. For other lymphoproliferative disorders apart of follicular lymphoma, scientists have discovered other strong protective associations like HLA-DRB1*04:01 for patients with DLBCL [20].

The study revealed novel correlations between chronic lymphoproliferative disorders and specific HLA alleles; however, it is important to acknowledge various limitations. One primary constraint refers to the small number of patients. However, the preliminary data we report here are new and interesting and will be the basis for future extended studies. Another limitation has to take into account the intrinsic polymorphism of HLA genes which makes a definite association between these genes and specific diseases a challenge. Another issue that needs to be addressed in the future is a better statistical match for gender and age groups.

## 5. Conclusions

Our preliminary data indicate that HLA-C*02:02 and HLA-C*12:02 alleles are positively linked to chronic lymphoproliferative disorders in Romanian patients. Also, HLA-A*11:01, HLA-B*35:02, HLA-B*81:01, HLA-DRB1*11:01, and HLA-DRB1*13:02 expression could indicate a lower risk of chronic lymphoproliferative disorders development. HLA and disease association is a very promising research field, expanding our understanding of the genetic factors affecting immunity. Overall, the present work demonstrates an evident statistical associations between HLA genes and chronic lymphoproliferative disorders, underlying the role of HLA genotyping when understanding the immune response in CLD.

## Figures and Tables

**Table 1:** Case and controls demographics.

	Total	Gender		Median Age
		Male (%)	Female (%)	
All patients	38	25	13	38
PTCL-NOS	16	11	5	50
Burkitt lymphoma	5	3	2	36
DLBCL	6	4	2	44
ATLL	4	3	1	48
Primary cutaneous γδ T-cell lymphoma	2	0	2	55
Mantle cell lymphoma	3	3	0	53
Mycosis fungoides	1	1	0	29
Sézary syndrome	1	0	1	50
Controls	50	28	22	34

Chronic lymphoproliferative diseases: PTCL-NOS (Peripheral T-cell lymphoma not otherwise specified), Burkitt lymphoma, DLBCL (diffuse large B-cell lymphoma), ATLL (adult T-cell lymphoma), primary cutaneous γδ T-cell lymphoma, mantle cell lymphoma, mycosis fungoides, Sézary syndrome.

**Table 2:** Distribution of HLA alleles in patients with chronic lymphoproliferative disorders and the control group. Comparison of the most important HLA alleles at the 4-digit level between patients and the control group.

Alleles	Cases n1 = 76	Controls n2 = 100	*p*-Value	OR	95% Confidence Interval	
	Number	Number			Low	Upper
HLA-A*11:01	9	2	0.010	0.169	0.038	0.759
HLA-B*35:02	0	6	0.037	0.940	0.895	0.988
HLA-B*81:01	0	6	0.037	0.940	0.895	0.988
HLA-C*02:02	7	0	0.002	1.101	1.025	1.183
HLA-C*07:02	11	5	0.036	0.345	0.125	0.952
HLA-C*12:02	7	0	0.002	1.101	1.025	1.183
HLA-DRB1*11:01	8	2	0.021	0.190	0.042	0.869
HLA-DRB1*13:02	0	6	0.037	0.940	0.895	0.988

Statistical significance was determined after calculating the *p*-value, OR (odds ratio), and CI (confidence interval). The Chi-square test or Fisher’s test was used to estimate the differences between the patient and control groups; *n*: number of alleles in the patient and control groups. A complete list of alleles associated with the disease is provided in Supplementary Table S1.

**Table 3:** Distribution of the HLA alleles in patients with different chronic lymphoproliferative disorders and the control group. Comparison of the most important HLA alleles at the 4-digit level between patients and the control group.

Disease	Allele	Cases n1 = 76	Controls n2 = 100	*p*-Value	OR	95% Confidence Interval	
		Number	Number			Low	Upper
PTLC-NOS	HLA-A*11:01	5	2	0.009	0.128	0.026	0.628
PTLC-NOS	HLA-C*12:02	6	0	0.0001	1.231	1.042	1.454
DLBCL	HLA-B*39:01	2	1	0.03	0.060	0.006	0.613
Burkitt lymphoma	HLA-C*06:02	3	7	0.047	0.233	0.071	0.764

Statistical significance was determined after calculating the *p*-value, OR (odds ratio), and CI (confidence interval). The Chi-square test or Fisher’s test was used to estimate the differences between the patient and control groups; *n*: number of alleles in the patient and control groups. A complete list of alleles associated with the disease is provided in Supplementary Tables S2 and S3. Abbreviations: peripheral T-cell lymphoma not otherwise specified (PTCL-NOS), Burkitt lymphoma, diffuse large B-cell lymphoma (DLBCL).

## Data Availability

Data available on request due to restrictions, e.g., privacy or ethical.

## Supplementary Materials

The following supporting information can be downloaded at: https://www.mdpi.com/article/10.3390/medsci12010014/s1. Table S1: Distribution of HLA alleles in patients with chronic lymphoproliferative disorders and the control group; Table S2: Distribution of HLA alleles in patients with Peripheral T-cell lymphoma not otherwise specified (PTCL-NOS) and the control group.; Table S3: Distribution of HLA alleles in patients with Diffuse large B-cell lymphoma (DLBCL) and the control group.
