# Supplementary material for: Immunogenetic Background of Chronic Lymphoproliferative Disorders in Romanian Patients—Case Control Study
Source: Med Sci (Basel). 2024 Feb 23;12(1):14. doi: 10.3390/medsci12010014 (PMC10972167; doi:10.3390/medsci12010014)
Supplement: Supplementary file 1 [file medsci-12-00014-s001.zip › Supplementary Table S1.pdf]

Supplemental Table S1. Distribution of HLA alleles in patients with chronic lymphoproliferative disorders and the control group. Comparison of the most important HLA alleles at the 4-digit level between patients and the control group.

**Allele**	**Cases n1=76**	**Controls n2=100**	**P-value**	**OR**	**95% Confidence interval**	
	number	number	Chi-square or **Fisher's** test		Low	Upper
**HLA-A*01:01**	9	14	0.674	1.182	0.541	2.585
**HLA-A*02:01**	21	24	0.584	0.869	0.525	1.438
**HLA-A*02:02**	0	2	0.506	0.980	0.953	1.008
**HLA-A*02:05**	1	0	0.432	1.013	0.987	1.040
**HLA-A*02:17**	1	0	0.432	1.013	0.987	1.040
**HLA-A*03:01**	4	12	0.185	2.280	0.765	6.792
**HLA-A*03:02**	1	0	0.432	1.013	0.987	1.040
**HLA-A*11:01**	9	2	0.010	0.169	0.038	0.759
**HLA-A*23:01**	1	0	0.432	1.013	0.987	1.040
**HLA-A*24:02**	11	6	0.059	0.415	0.160	1.071
**HLA-A*24:03**	1	0	0.432	1.013	0.987	1.040
**HLA-A*25:01**	2	4	0.700	1.520	0.286	8.082
**HLA-A*26:01**	4	3	0.467	0.570	0.131	2.471
**HLA-A*29:01**	0	1	1.000	0.990	0.971	1.010
**HLA-A*29:02**	0	4	0.135	0.960	0.922	.999
**HLA-A*30:01**	2	4	0.700	1.520	0.286	8.082
**HLA-A*30:02**	0	1	1.000	0.990	0.971	1.010
**HLA-A*30:04**	0	1	1.000	0.990	0.971	1.010
**HLA-A*31:01**	3	5	1.000	1.267	0.312	5.136
**HLA-A*32:01**	2	1	0.579	0.380	0.035	4.113
**HLA-A*33:01**	1	2	1.000	1.520	0.140	16.454
**HLA-A*33:03**	1	0	0.432	1.013	0.987	1.040
**HLA-A*66:01**	1	1	1.000	0.760	0.048	11.957
**HLA-A*66:01**	0	2	0.506	0.980	0.953	1.008

**HLA-A*68:01**	1	0	0.432	1.013	0.987	1.040
**HLA-A*68:02**	0	5	0.071	0.950	0.908	0.994
**HLA-B*07:02**	10	5	0.055	0.380	0.135	1.066
**HLA-B*08:01**	3	8	0.354	2.027	0.556	7.384
**HLA-B*13:02**	7	4	0.211	0.434	0.132	1.430
**HLA-B*14:01**	0	1	1.000	0.990	0.971	1.010
**HLA-B*14:02**	3	1	0.317	0.253	0.027	2.388
**HLA-B*15:01**	1	1	1.000	0.760	0.048	11.957
**HLA-B*15:10**	0	1	1.000	0.990	0.971	1.010
**HLA-B*18:01**	3	8	0.354	2.027	0.556	7.384
**HLA-B*18:03**	0	1	1.000	0.990	0.971	1.010
**HLA-B*18:04**	0	1	1.000	0.990	0.971	1.010
**HLA-B*18:05**	0	2	0.506	0.980	0.953	1.008
**HLA-B*27:02**	0	1	1.000	0.990	0.971	1.010
**HLA-B*27:05**	1	2	1.000	1.520	0.140	16.454
**HLA-B*35:01**	6	3	0.177	0.380	0.098	1.471
**HLA-B*35:02**	0	6	0.037	0.940	0.895	0.988
**HLA-B*35:03**	6	3	0.177	0.380	0.098	1.471
**HLA-B*35:08**	2	0	1.027	.990	1.066	1.027
**HLA-B*37:01**	0	1	1.000	0.990	0.971	1.010
**HLA-B*38:01**	3	1	0.315	0.250	0.027	2.356
**HLA-B*39:01**	4	1	0.167	0.190	0.022	1.666
**HLA-B*39:06**	1	0	0.432	1.013	0.987	1.040
**HLA-B*40:02**	1	4	0.391	3.040	0.347	26.650
**HLA-B*40:06**	0	1	1.000	0.990	0.971	1.010
**HLA-B*41:01**	1	3	0.635	2.280	0.242	21.490
**HLA-B*41:02**	1	2	1.000	1.520	0.140	16.454
**HLA-B*44:02**	0	4	0.135	0.960	0.922	.999
**HLA-B*44:03**	2	6	0.469	2.280	0.473	10.984
**HLA-B*49:01**	0	3	0.260	0.970	0.937	1.004
**HLA-B*50:01**	2	0	0.185	1.027	0.990	1.066

**HLA-B*51:01**	6	8	0.980	1.013	0.367	2.798
**HLA-B*52:01**	4	4	0.728	0.760	0.196	2.942
**HLA-B*55:01**	1	4	0.391	3.040	0.347	26.650
**HLA-B*56:01**	1	0	0.432	1.013	0.987	1.040
**HLA-B*57:01**	2	1	0.579	0.380	0.035	4.113
**HLA-B*58:01**	3	1	0.317	0.253	0.027	2.388
**HLA-B*58:02**	0	1	1.000	0.990	0.971	1.010
**HLA-B*59:01**	0	1	1.000	0.990	0.971	1.010
**HLA-B*81:01**	0	6	0.037	0.940	0.895	0.988
**HLA-C*01:02**	4	7	0.759	1.330	0.404	4.379
**HLA-C*02:02**	7	0	0.002	1.101	1.025	1.183
**HLA-C*03:03**	1	2	1.000	1.520	0.140	16.454
**HLA-C*03:04**	1	1	1.000	0.760	0.048	11.957
**HLA-C*04:01**	10	18	0.414	1.368	0.670	2.791
**HLA-C*05:01**	0	2	0.506	0.980	0.953	1.008
**HLA-C*06:02**	9	7	0.268	0.591	0.231	1.516
**HLA-C*07:01**	7	15	0.358	1.629	0.699	3.796
**HLA-C*07:02**	11	5	0.036	0.345	0.125	0.952
**HLA-C*07:04**	0	1	1.000	0.990	0.971	1.010
**HLA-C*08:02**	4	2	0.405	0.380	0.071	2.021
**HLA-C*12:02**	7	0	0.002	1.101	1.025	1.183
**HLA-C*12:03**	7	8	0.791	0.869	0.329	2.290
**HLA-C*12:12**	0	1	1.000	0.990	0.971	1.010
**HLA-C*14:02**	1	0	0.432	1.013	0.987	1.040
**HLA-C*15:02**	4	2	0.405	0.380	0.071	2.021
**HLA-C*15:04**	1	0	0.432	1.013	0.987	1.040
**HLA-C*15:05**	1	0	0.432	1.013	0.987	1.040
**HLA-C*15:13**	0	2	0.506	0.980	0.953	1.008
**HLA-C*16:01**	0	4	0.135	0.960	0.922	0.999
**HLA-C*16:02**	0	1	1.000	0.990	0.971	1.010
**HLA-C*16:04**	0	1	1.000	0.990	0.971	1.010

**HLA-C*17:01**	0	2	0.506	0.980	0.953	1.008
**HLA-C*17:03**	1	3	0.635	2.280	0.242	21.490
**HLA-C*18:01**	0	1	1.000	0.990	0.971	1.010
**HLA-DPB1*01:01**	1	9	0.045	6.840	0.886	52.833
**HLA-DPB1*02:01**	4	14	0.078	2.660	0.912	7.758
**HLA-DPB1*02:02**	0	1	1.000	0.990	0.971	1.010
**HLA-DPB1*03:01**	6	10	0.793	1.267	0.482	3.332
**HLA-DPB1*04:01**	19	31	0.404	1.240	0.762	2.018
**HLA-DPB1*04:02**	12	15	1.000	0.950	0.473	1.909
**HLA-DPB1*05:01**	0	4	0.135	0.960	0.922	0.999
**HLA-DPB1*09:01**	2	2	1.000	0.760	0.110	5.274
**HLA-DPB1*10:01**	0	3	0.260	0.970	0.937	1.004
**HLA-DPB1*13:01**	2	1	0.579	0.380	0.035	4.113
**HLA-DPB1*14:01**	0	1	1.000	0.990	0.971	1.010
**HLA-DPB1*17:01**	0	3	0.260	0.970	0.937	1.004
**HLA-DPB1*18:01**	0	1	1.000	0.990	0.971	1.010
**HLA-DPB1*23:01**	0	1	1.000	0.990	0.971	1.010
**HLA-DPB1*104:01**	0	3	0.260	0.970	0.937	1.004
**HLA-DPB1*105:01**	0	1	1.000	0.990	0.971	1.010
**HLA-DQB1*02:01**	7	12	0.555	1.303	0.539	3.151
**HLA-DQB1*02:02**	7	10	1.000	1.086	0.433	2.721
**HLA-DQB1*03:01**	18	21	0.716	0.887	0.509	1.544
**HLA-DQB1*03:02**	5	3	0.294	0.456	0.112	1.849
**HLA-DQB1*03:03**	1	0	0.432	1.013	0.987	1.040
**HLA-DQB1*03:19**	0	1	1.000	0.990	0.971	1.010
**HLA-DQB1*04:02**	2	3	1.000	1.140	0.195	6.654
**HLA-DQB1*05:01**	7	9	0.962	0.977	0.381	2.506
**HLA-DQB1*05:02**	12	14	0.740	0.887	0.435	1.805
**HLA-DQB1*05:03**	1	5	0.237	3.800	0.453	31.856
**HLA-DQB1*05:04**	0	1	1.000	0.990	0.971	1.010

**HLA-DQB1*06:01**	4	2	0.405	0.380	0.071	2.021
**HLA-DQB1*06:02**	6	7	0.822	0.887	0.311	2.531
**HLA-DQB1*06:03**	5	5	0.654	0.760	0.228	2.531
**HLA-DQB1*06:04**	1	3	2.280	0.242	21.490	2.280
**HLA-DQB1*06:09**	0	1	1.000	0.990	0.971	1.010
**HLA-DRB1*01:01**	4	7	0.759	1.330	0.404	4.379
**HLA-DRB1*01:02**	1	0	0.432	1.013	0.987	1.040
**HLA-DRB1*03:01**	7	13	0.481	1.411	0.592	3.366
**HLA-DRB1*03:02**	0	1	1.000	0.990	0.971	1.010
**HLA-DRB1*04:01**	1	3	2.280	0.242	21.490	2.280
**HLA-DRB1*04:02**	1	0	0.432	1.013	0.987	1.040
**HLA-DRB1*04:04**	3	0	0.079	1.041	0.995	1.090
**HLA-DRB1*04:05**	0	1	1.000	0.990	0.971	1.010
**HLA-DRB1*04:07**	1	0	0.432	1.013	0.987	1.040
**HLA-DRB1*07:01**	8	13	1.235	0.539	2.829	1.235
**HLA-DRB1*08:01**	1	2	1.000	1.520	0.140	16.454
**HLA-DRB1*10:01**	0	2	0.506	0.980	0.953	1.008
**HLA-DRB1*11:01**	8	2	0.021	0.190	0.042	.869
**HLA-DRB1*11:02**	0	2	0.506	0.980	0.953	1.008
**HLA-DRB1*11:03**	1	1	1.000	0.760	0.048	11.957
**HLA-DRB1*11:04**	7	11	0.804	1.194	0.486	2.936
**HLA-DRB1*12:01**	1	2	1.000	1.520	0.140	16.454
**HLA-DRB1*13:01**	6	5	0.534	0.633	0.201	1.998
**HLA-DRB1*13:02**	0	6	0.037	0.940	0.895	0.988
**HLA-DRB1*13:03**	2	3	1.000	1.140	0.195	6.654
**HLA-DRB1*13:05**	0	1	1.000	0.990	0.971	1.010
**HLA-DRB1*14:01**	0	1	1.000	0.990	0.971	1.010
**HLA-DRB1*14:04**	0	1	1.000	0.990	0.971	1.010
**HLA-DRB1*14:54**	1	4	0.391	3.040	0.347	26.650
**HLA-DRB1*15:01**	6	7	0.822	0.887	0.311	2.531
**HLA-DRB1*15:02**	4	3	0.467	0.570	0.131	2.471

**HLA-DRB1*16:01**	11	7	0.105	0.484	0.197	1.189
**HLA-DRB1*16:02**	2	1	0.579	0.380	0.035	4.113

* Statistical significance was determined after calculating the *p*-value, OR (odds ratio), and CI (confidence interval). The chi-square test or Fisher's test was used to estimate the differences between the patient and control groups; *n*: number of alleles in the patient and control groups
