# Supplementary material for: Immunogenetic Background of Chronic Lymphoproliferative Disorders in Romanian Patients—Case Control Study
Source: Med Sci (Basel). 2024 Feb 23;12(1):14. doi: 10.3390/medsci12010014 (PMC10972167; doi:10.3390/medsci12010014)
Supplement: Supplementary file 1 [file medsci-12-00014-s001.zip › Supplementary Table S2.pdf]

Supplemental Table S2. Distribution of HLA alleles in patients with Peripheral T-cell lymphoma not otherwise specified (PTCL-NOS) and the control group. Comparison of the most important HLA alleles at the 4-digit level between patients and the control group.

**Allele**	**Cases** n1=32	**Controls** n2=100	**P-value**	**OR**	**95% Confidence interval**	
	number	number	Chi-square or **Fisher's** test		Low	Upper
**HLA-A*01:01**	6	14	.514	.747	.313	1.781
**HLA-A*02:01**	7	24	.805	1.097	.522	2.304
**HLA-A*02:02**	0	2	1.000	.980	.953	1.008
**HLA-A*03:01**	2	12	.516	1.920	.454	8.128
**HLA-A*11:01**	5	2	.009	.128	.026	.628
**HLA-A*23:01**	1	0	.242	1.032	.970	1.099
**HLA-A*24:02**	4	6	.255	.480	.144	1.595
**HLA-A*25:01**	0	4	.572	.960	.922	.999
**HLA-A*26:01**	1	3	1.000	.960	.103	8.909
**HLA-A*29:01**	0	1	1.000	.990	.971	1.010
**HLA-A*29:02**	0	4	.572	.960	.922	.999
**HLA-A*30:01**	1	4	1.000	1.280	.148	11.042
**HLA-A*30:02**	0	1	1.000	.990	.971	1.010
**HLA-A*30:04**	0	1	1.000	.990	.971	1.010
**HLA-A*31:01**	1	5	1.000	1.600	.194	13.195
**HLA-A*32:01**	0	1	1.000	.990	.971	1.010
**HLA-A*33:01**	1	2	.568	.640	.060	6.827
**HLA-A*33:03**	1	0	.242	1.032	.970	1.099
**HLA-A*66:01**	1	1	.427	.320	.021	4.971
**HLA-A*66:02**	0	2	1.000	.980	.953	1.008
**HLA-A*68:01**	1	0	.242	1.032	.970	1.099
**HLA-A*68:02**	0	5	0.335	.950	.908	.994

**HLA-B*07:02**	2	5	.676	.800	.163	3.926
**HLA-B*08:01**	2	8	1.000	1.280	.286	5.722
**HLA-B*13:02**	4	4	.097	1.097	.957	1.258
**HLA-B*14:01**	0	1	1.000	.990	.971	1.010
**HLA-B*14:02**	2	1	.146	.160	.015	1.707
**HLA-B*15:01**	0	1	1.000	.990	.971	1.010
**HLA-B*15:10**	0	1	1.000	.990	.971	1.010
**HLA-B*18:01**	2	8	1.000	1.280	.286	5.722
**HLA-B*18:03**	0	1	1.000	.990	.971	1.010
**HLA-B*18:04**	0	1	1.000	.990	.971	1.010
**HLA-B*18:05**	0	2	1.000	.980	.953	1.008
**HLA-B*27:02**	0	1	1.000	.990	.971	1.010
**HLA-B*27:05**	0	2	1.000	.980	.953	1.008
**HLA-B*35:01**	3	3	.153	.320	.068	1.508
**HLA-B*35:02**	0	6	.335	.940	.895	.988
**HLA-B*35:03**	3	3	.153	.320	.068	1.508
**HLA-B*35:08**	2	0	1.067	.975	1.166	1.067
**HLA-B*37:01**	0	1	1.000	.990	.971	1.010
**HLA-B*38:01**	0	1	1.000	.990	.971	1.010
**HLA-B*39:01**	0	1	1.000	.990	.971	1.010
**HLA-B*39:06**	1	0	.242	1.032	.970	1.099
**HLA-B*40:02**	0	4	.572	.960	.922	.999
**HLA-B*40:06**	0	1	1.000	.990	.971	1.010
**HLA-B*41:01**	0	3	1.000	.970	.937	1.004
**HLA-B*41:02**	1	2	.568	.640	.060	6.827
**HLA-B*44:02**	0	4	.572	.960	.922	.999
**HLA-B*44:03**	1	6	1.000	1.920	.240	15.357
**HLA-B*49:01**	0	3	1.000	.970	.937	1.004
**HLA-B*51:01**	0	8	.198	.920	.868	.975

**HLA-B*52:01**	3	4	.359	.427	.101	1.806
**HLA-B*55:01**	1	4	1.000	1.280	.148	11.042
**HLA-B*56:01**	1	0	.242	1.032	.970	1.099
**HLA-B*57:01**	0	1	1.000	.990	.971	1.010
**HLA-B*58:01**	2	1	.146	.160	.015	1.707
**HLA-B*58:02**	0	1	1.000	.990	.971	1.010
**HLA-B*59:01**	0	1	1.000	.990	.971	1.010
**HLA-B*81:01**	0	6	.335	.940	.895	.988
**HLA-C*01:02**	3	7	.704	.747	.205	2.720
**HLA-C*02:02**	1	0	.242	1.032	.970	1.099
**HLA-C*03:03**	0	2	1.000	.980	.953	1.008
**HLA-C*03:04**	1	1	.427	.320	.021	4.971
**HLA-C*04:01**	6	18	.924	.960	.417	2.210
**HLA-C*05:01**	0	2	1.000	.980	.953	1.008
**HLA-C*06:02**	4	7	.461	.560	.175	1.790
**HLA-C*07:01**	3	15	.560	1.600	.495	5.176
**HLA-C*07:02**	3	5	.401	.533	.135	2.109
**HLA-C*07:04**	0	1	1.000	.990	.971	1.010
**HLA-C*08:02**	2	2	.247	.320	.047	2.181
**HLA-C*12:02**	6	0	.0001	1.231	1.042	1.454
**HLA-C*12:03**	1	8	.687	2.560	.333	19.696
**HLA-C*12:12**	0	1	1.000	.990	.971	1.010
**HLA-C*15:02**	1	2	.568	.640	.060	6.827
**HLA-C*15:13**	0	2	1.000	.980	.953	1.008
**HLA-C*16:01**	0	4	.572	.960	.922	.999
**HLA-C*16:02**	0	1	1.000	.990	.971	1.010
**HLA-C*16:04**	0	1	1.000	.990	.971	1.010
**HLA-C*17:01**	0	2	1.000	.980	.953	1.008
**HLA-C*17:03**	1	3	1.000	.960	.103	8.909

**HLA-C*18:01**	0	1	1.000	.990	.971	1.010
**HLA-DPB1*01:01**	0	9	.133	.910	.856	.968
**HLA-DPB1*02:01**	2	14	.355	2.240	.538	9.333
**HLA-DPB1*02:02**	0	1	1.000	.990	.971	1.010
**HLA-DPB1*03:01**	5	10	.358	.640	.236	1.734
**HLA-DPB1*04:01**	7	31	.376	1.417	.692	2.903
**HLA-DPB1*04:02**	5	15	.932	.960	.379	2.434
**HLA-DPB1*05:01**	0	4	.572	.960	.922	.999
**HLA-DPB1*09:01**	1	2	.568	.640	.060	6.827
**HLA-DPB1*10:01**	0	3	1.000	.970	.937	1.004
**HLA-DPB1*13:01**	0	1	1.000	.990	.971	1.010
**HLA-DPB1*14:01**	0	1	1.000	.990	.971	1.010
**HLA-DPB1*17:01**	0	3	1.000	.970	.937	1.004
**HLA-DPB1*18:01**	0	1	1.000	.990	.971	1.010
**HLA-DPB1*23:01**	0	1	1.000	.990	.971	1.010
**HLA-DPB1*104:01**	0	3	1.000	.970	.937	1.004
**HLA-DPB1*105:01**	0	1	1.000	.990	.971	1.010
**HLA-DQB1*02:01**	3	12	1.000	1.280	.385	4.254
**HLA-DQB1*02:02**	5	10	.358	1.667	.524	5.298
**HLA-DQB1*03:01**	8	21	.630	.840	.413	1.709
**HLA-DQB1*03:02**	1	3	1.000	.960	.103	8.909
**HLA-DQB1*03:19**	0	1	1.000	.990	.971	1.010
**HLA-DQB1*04:02**	0	3	1.000	.970	.937	1.004
**HLA-DQB1*05:01**	3	9	1.000	.960	.277	3.332
**HLA-DQB1*05:02**	5	14	.779	.896	.350	2.295
**HLA-DQB1*05:03**	1	5	1.000	1.600	.194	13.195
**HLA-DQB1*05:04**	0	1	1.000	.990	.971	1.010

**HLA-DQB1*06:01**	3	2	.092	.213	.037	1.221
**HLA-DQB1*06:02**	1	7	.679	2.240	.286	17.525
**HLA-DQB1*06:03**	1	5	1.000	1.600	.194	13.195
**HLA-DQB1*06:04**	1	3	1.000	.960	.103	8.909
**HLA-DQB1*06:09**	0	1	1.000	.990	.971	1.010
**HLA-DRB1*01:01**	1	7	.679	2.240	.286	17.525
**HLA-DRB1*01:02**	1	0	.242	1.032	.970	1.099
**HLA-DRB1*03:01**	3	13	.760	1.387	.422	4.561
**HLA-DRB1*03:02**	0	1	1.000	.990	.971	1.010
**HLA-DRB1*04:01**	0	3	1.000	.970	.937	1.004
**HLA-DRB1*04:04**	2	0	.057	1.067	.975	1.166
**HLA-DRB1*04:05**	0	1	1.000	.990	.971	1.010
**HLA-DRB1*07:01**	5	13	.769	.832	.321	2.155
**HLA-DRB1*08:01**	0	2	1.000	.980	.953	1.008
**HLA-DRB1*10:01**	0	2	1.000	.980	.953	1.008
**HLA-DRB1*11:01**	4	2	.03	.160	.031	.833
**HLA-DRB1*11:02**	0	2	1.000	.980	.953	1.008
**HLA-DRB1*11:03**	0	1	1.000	.990	.971	1.010
**HLA-DRB1*11:04**	2	11	.733	1.760	.412	7.526
**HLA-DRB1*12:01**	1	2	.568	.640	.060	6.827
**HLA-DRB1*13:01**	1	5	1.000	1.600	.194	13.195
**HLA-DRB1*13:02**	0	6	.335	.940	.895	.988
**HLA-DRB1*13:03**	1	3	1.000	.960	.103	8.909
**HLA-DRB1*13:05**	0	1	1.000	.990	.971	1.010
**HLA-DRB1*14:01**	0	1	1.000	.990	.971	1.010
**HLA-DRB1*14:04**	0	1	1.000	.990	.971	1.010
**HLA-DRB1*14:54**	1	4	1.000	1.280	.148	11.042
**HLA-DRB1*15:01**	0	7	.194	.930	.881	.981
**HLA-DRB1*15:02**	4	3	.059	.240	.057	1.016

**HLA-DRB1*16:01**	5	7	.162	.448	.153	1.314
**HLA-DRB1*16:02**	1	1	.427	.320	.021	4.971

* Statistical significance was determined after calculating the *p*-value, OR (odds ratio), and CI (confidence interval). The chi-square test or Fisher's test was used to estimate the differences between the patient and control groups; *n*: number of alleles in the patient and control groups.
