# Supplementary material for: Immunogenetic Background of Chronic Lymphoproliferative Disorders in Romanian Patients—Case Control Study
Source: Med Sci (Basel). 2024 Feb 23;12(1):14. doi: 10.3390/medsci12010014 (PMC10972167; doi:10.3390/medsci12010014)
Supplement: Supplementary file 1 [file medsci-12-00014-s001.zip › Supplementary Table S3.pdf]

Supplemental Table S3. Distribution of HLA alleles in patients with Diffuse large B-cell lymphoma (DLBCL). and the control group. Comparison of the most important HLA alleles at the 4-digit level between patients and the control group.

Allele	Cases n1=12	Controls n2=100	P-value	OR	95% Confidence interval	
	number	number	Chi-square or **Fisher's** test		Low	Upper
**HLA-A*01:01**	1	14	1.000	1.680	.242	11.672
**HLA-A*02:01**	3	24	1.000	.960	.339	2.717
**HLA-A*02:02**	0	2	1.000	.980	.953	1.008
**HLA-A*02:05**	1	0	.107	1.091	.920	1.294
**HLA-A*03:01**	1	12	1.000	1.440	.205	10.123
**HLA-A*11:01**	0	2	1.000	.980	.953	1.008
**HLA-A*24:02**	3	6	.055	.240	.069	.838
**HLA-A*25:01**	1	4	.439	.480	.058	3.951
**HLA-A*26:01**	1	3	.369	.360	.041	3.193
**HLA-A*29:01**	0	1	1.000	.990	.971	1.010
**HLA-A*29:02**	0	4	1.000	.960	.922	.999
**HLA-A*30:01**	0	4	1.000	.960	.922	.999
**HLA-A*30:02**	0	1	1.000	.990	.971	1.010
**HLA-A*30:04**	0	1	1.000	.990	.971	1.010
**HLA-A*31:01**	0	5	1.000	.950	.908	.994
**HLA-A*32:01**	1	1	.204	.120	.008	1.797
**HLA-A*33:01**	0	2	1.000	.980	.953	1.008
**HLA-A*66:01**	0	1	1.000	.990	.971	1.010
**HLA-A*66:02**	0	2	1.000	.980	.953	1.008
**HLA-A*68:02**	0	5	1.000	.950	.908	.994
**HLA-B*07:02**	2	5	.163	.300	.065	1.381
**HLA-B*08:01**	0	8	.596	.920	.868	.975

**HLA-B*13:02**	0	4	1.000	.960	.922	.999
**HLA-B*14:01**	0	1	1.000	.990	.971	1.010
**HLA-B*14:02**	0	1	1.000	.990	.971	1.010
**HLA-B*15:01**	0	1	1.000	.990	.971	1.010
**HLA-B*15:10**	0	1	1.000	.990	.971	1.010
**HLA-B*18:01**	1	8	1.000	.960	.131	7.028
**HLA-B*18:03**	0	1	1.000	.990	.971	1.010
**HLA-B*18:04**	0	1	1.000	.990	.971	1.010
**HLA-B*18:05**	0	2	1.000	.980	.953	1.008
**HLA-B*27:02**	0	1	1.000	.990	.971	1.010
**HLA-B*27:05**	0	2	1.000	.980	.953	1.008
**HLA-B*35:01**	2	3	.088	.180	.033	.972
**HLA-B*35:02**	0	6	1.000	.940	.895	.988
**HLA-B*35:03**	0	3	1.000	.970	.937	1.004
**HLA-B*37:01**	0	1	1.000	.990	.971	1.010
**HLA-B*38:01**	2	1	.030	.060	.006	.613
**HLA-B*39:01**	2	1	.030	.060	.006	.613
**HLA-B*40:02**	0	4	1.000	.960	.922	.999
**HLA-B*40:06**	0	1	1.000	.990	.971	1.010
**HLA-B*41:01**	0	3	1.000	.970	.937	1.004
**HLA-B*41:02**	0	2	1.000	.980	.953	1.008
**HLA-B*44:02**	0	4	1.000	.960	.922	.999
**HLA-B*44:03**	0	6	1.000	.940	.895	.988
**HLA-B*49:01**	0	3	1.000	.970	.937	1.004
**HLA-B*51:01**	2	8	.291	.480	.115	2.004
**HLA-B*52:01**	0	4	1.000	.960	.922	.999
**HLA-B*55:01**	0	4	1.000	.960	.922	.999
**HLA-B*57:01**	1	1	.204	.120	.008	1.797
**HLA-B*58:01**	0	1	1.000	.990	.971	1.010

**HLA-B*58:02**	0	1	1.000	.990	.971	1.010
**HLA-B*59:01**	0	1	1.000	.990	.971	1.010
**HLA-B*81:01**	0	6	1.000	.940	.895	.988
**HLA-C*01:02**	1	7	1.000	.840	.113	6.256
**HLA-C*02:02**	1	0	.107	1.091	.920	1.294
**HLA-C*03:03**	0	2	1.000	.980	.953	1.008
**HLA-C*03:04**	0	1	1.000	.990	.971	1.010
**HLA-C*04:01**	2	18	1.000	1.080	.285	4.094
**HLA-C*05:01**	0	2	1.000	.980	.953	1.008
**HLA-C*06:02**	1	7	1.000	.840	.113	6.256
**HLA-C*07:01**	2	15	1.000	.900	.234	3.466
**HLA-C*07:02**	1	5	.502	.600	.076	4.716
**HLA-C*07:04**	0	1	1.000	.990	.971	1.010
**HLA-C*08:02**	1	2	.291	.240	.023	2.453
**HLA-C*12:03**	3	8	.095	.320	.098	1.046
**HLA-C*12:12**	0	1	1.000	.990	.971	1.010
**HLA-C*15:02**	0	2	1.000	.980	.953	1.008
**HLA-C*15:13**	0	2	1.000	.980	.953	1.008
**HLA-C*16:01**	0	4	1.000	.960	.922	.999
**HLA-C*16:02**	0	1	1.000	.990	.971	1.010
**HLA-C*16:04**	0	1	1.000	.990	.971	1.010
**HLA-C*17:01**	0	2	1.000	.980	.953	1.008
**HLA-C*17:03**	0	3	1.000	.970	.937	1.004
**HLA-C*18:01**	0	1	1.000	.990	.971	1.010
**HLA-DPB1*01:01**	0	9	.594	.910	.856	.968
**HLA-DPB1*02:01**	1	14	1.000	.938	.777	1.132
**HLA-DPB1*02:02**	0	1	1.000	.990	.971	1.010
**HLA-DPB1*03:01**	0	10	.597	.900	.843	.961
**HLA-DPB1*04:01**	3	31	1.000	1.240	.446	3.448

**HLA-DPB1*04:02**	4	15	.120	.450	.178	1.136
**HLA-DPB1*05:01**	0	4	1.000	.960	.922	.999
**HLA-DPB1*09:01**	0	2	1.000	.980	.953	1.008
**HLA-DPB1*10:01**	0	3	1.000	.970	.937	1.004
**HLA-DPB1*13:01**	0	1	1.000	.990	.971	1.010
**HLA-DPB1*14:01**	0	1	1.000	.990	.971	1.010
**HLA-DPB1*17:01**	0	3	1.000	.970	.937	1.004
**HLA-DPB1*18:01**	0	1	1.000	.990	.971	1.010
**HLA-DPB1*23:01**	0	1	1.000	.990	.971	1.010
**HLA-DPB1*104:01**	0	3	1.000	.970	.937	1.004
**HLA-DPB1*105:01**	0	1	1.000	.990	.971	1.010
**HLA-DQB1*02:01**	0	12	.357	.880	.819	.946
**HLA-DQB1*02:02**	0	10	.597	.900	.843	.961
**HLA-DQB1*03:01**	4	21	.461	.630	.260	1.528
**HLA-DQB1*03:02**	2	3	.088	.180	.033	.972
**HLA-DQB1*03:19**	0	1	1.000	.990	.971	1.010
**HLA-DQB1*04:02**	1	3	.369	.360	.041	3.193
**HLA-DQB1*05:01**	1	9	1.000	1.080	.150	7.801
**HLA-DQB1*05:02**	2	14	.681	.840	.217	3.257
**HLA-DQB1*05:03**	0	5	1.000	.950	.908	.994
**HLA-DQB1*05:04**	0	1	1.000	.990	.971	1.010
**HLA-DQB1*06:01**	1	2	.291	.240	.023	2.453
**HLA-DQB1*06:02**	0	7	1.000	.930	.881	.981
**HLA-DQB1*06:03**	1	5	.502	.600	.076	4.716
**HLA-DQB1*06:04**	0	3	1.000	.970	.937	1.004
**HLA-DQB1*06:09**	0	1	1.000	.990	.971	1.010
**HLA-DRB1*01:01**	0	7	1.000	.930	.881	.981

**HLA-DRB1*03:01**	0	13	.354	.870	.807	.938
**HLA-DRB1*03:02**	0	1	1.000	.990	.971	1.010
**HLA-DRB1*04:01**	1	3	.369	.360	.041	3.193
**HLA-DRB1*04:04**	1	0	.107	1.091	.920	1.294
**HLA-DRB1*04:05**	0	1	1.000	.990	.971	1.010
**HLA-DRB1*07:01**	0	13	.354	.870	.807	.938
**HLA-DRB1*08:01**	1	2	.291	.240	.023	2.453
**HLA-DRB1*10:01**	0	2	1.000	.980	.953	1.008
**HLA-DRB1*11:01**	2	2	.056	.120	.019	.776
**HLA-DRB1*11:02**	0	2	1.000	.980	.953	1.008
**HLA-DRB1*11:03**	1	1	.204	.120	.008	1.797
**HLA-DRB1*11:04**	2	11	.629	.660	.166	2.630
**HLA-DRB1*12:01**	0	2	1.000	.980	.953	1.008
**HLA-DRB1*13:01**	2	5	.163	.300	.065	1.381
**HLA-DRB1*13:02**	0	6	1.000	.940	.895	.988
**HLA-DRB1*13:03**	0	3	1.000	.970	.937	1.004
**HLA-DRB1*13:05**	0	1	1.000	.990	.971	1.010
**HLA-DRB1*14:01**	0	1	1.000	.990	.971	1.010
**HLA-DRB1*14:04**	0	1	1.000	.990	.971	1.010
**HLA-DRB1*14:54**	0	4	1.000	.960	.922	.999
**HLA-DRB1*15:01**	1	7	1.000	.840	.113	6.256
**HLA-DRB1*15:02**	0	3	1.000	.970	.937	1.004
**HLA-DRB1*16:01**	1	7	1.000	.840	.113	6.256
**HLA-DRB1*16:02**	0	1	1.000	.990	.971	1.010

* Statistical significance was determined after calculating the *p*-value, OR (odds ratio), and CI (confidence interval). The chi-square test or Fisher's test was used to estimate the differences between the patient and control groups; *n*: number of alleles in the patient and control groups.
